# Pseudoginsenoside-F11: a comprehensive review of chemical structure, pharmacological activities, pharmacokinetics, and therapeutic potential

**DOI:** 10.3389/fphar.2026.1808278

**Published:** 2026-05-28

**Authors:** Chao Wu, Xiaojuan Cong, Yuan Gao, Jinkun Liu, Yuanyuan Duan, Hongxia Yu, Shuai Gao, Wei Zhang

**Affiliations:** 1 Shandong Drug and Food Vocational College, Weihai, China; 2 Marine College, Shandong University, Weihai, China; 3 Shandong AnRan Nanometer Industry Development CO., Ltd., Weihai, China; 4 Weihai (Wendeng) Authentic Ginseng Industry Development Co., Ltd., Weihai, China

**Keywords:** chemical structure, neuroprotection, pharmacokinetics, pharmacological activities, pseudoginsenoside-F11

## Abstract

Pseudoginsenoside-F11 (PF11), an ocotillol-type saponin unique to American ginseng (*Panax quinquefolius* L.), is characterized by a distinctive 20,24-epoxy bridge. This review summarizes the current evidence regarding PF11’s chemical structure, pharmacological effects, pharmacokinetics, and therapeutic potential. Preclinical studies have demonstrated that PF11 exerts multi-targeted neuroprotective effects in animal models of Alzheimer’s disease, Parkinson’s disease, and cerebral ischemia. These neuroprotective actions are mediated through the regulation of calcium homeostasis, restoration of the autophagy-lysosomal pathway, and modulation of microglial polarization. Beyond neuroprotection, PF11 offers additional beneficial effects: it protects the heart by antagonizing the β1-adrenoceptor, safeguards the kidneys via inhibition of the NF-κB/NLRP3 pathway, regulates metabolism as a partial PPARγ agonist, and mitigates tolerance to morphine and methamphetamine. Despite promising preclinical evidence, PF11 remains at an early translational stage due to poor oral bioavailability, incomplete pharmacokinetic characterization, and the absence of human studies. Addressing these limitations through formulation optimization, mechanistic validation, and rigorous clinical investigation will determine whether PF11 can progress from a natural product lead to a viable therapeutic candidate.

## 1 Introduction

Ginseng, obtained from plants of the genus *Panax* (family Araliaceae), has been used in traditional medicine for thousands of years, particularly in Asian countries, and is valued for its adaptogenic and health-enhancing properties (Arring et al., 2018; Zhou et al., 2023). Its pharmacological activities are primarily attributed to ginsenosides, a class of triterpene saponins that serve as the primary bioactive components (Fan et al., 2020; Paik et al., 2023). Classified by their aglycone structures, ginsenosides fall into three main categories: dammarane-type, oleanane-type, and ocotillol-type compounds (Dou et al., 2024; Li et al., 2016). Among these, ocotillol-type ginsenosides form a unique subclass, predominantly present in American ginseng (Liu et al., 2012; Shi et al., 2012).

PF11 is a typical ocotillol-type ginsenoside and acts as a characteristic marker to distinguish American ginseng from Asian ginseng varieties (Chen et al., 2015; Kim et al., 2019). This triterpenoid saponin features a rigid four-ring dammarane skeleton with a distinct 20,24-epoxy bridge structure (Meng et al., 2010). PF11’s unique structural features endow it with specific pharmacological properties that set it apart from the dammarane-type ginsenosides prevalent in Asian ginseng. Over the past two decades, growing scientific evidence has demonstrated PF11’s significant therapeutic potential across multiple organ systems. Early studies revealed its ability to antagonize the behavioral effects of psychoactive substances such as morphine, methamphetamine, and scopolamine (Li et al., 1999; Li et al., 2000; Wu et al., 2003). Subsequent extensive research has clarified its neuroprotective effects in various neurodegenerative disease models, including Alzheimer’s disease (AD) and Parkinson’s disease (PD) (Chu et al., 2021; González-Burgos et al., 2015). More recent investigations have expanded the scope of PF11 research to include organ protection, cardiovascular protection, and tissue regeneration (Hou et al., 2020; Wang et al., 2024b; Zhang et al., 2023; Zhou et al., 2021).

Despite these promising findings, several critical knowledge gaps persist. The exact molecular mechanisms underlying PF11’s pleiotropic effects require further clarification, and its pharmacokinetic properties, including absorption, distribution, metabolism, and excretion, remain insufficiently characterized. Additionally, the compound’s poor oral bioavailability poses a major barrier to clinical development. To the best of our knowledge, this is the first comprehensive review dedicated to summarizing the chemical structure, pharmacological activities, pharmacokinetics, and therapeutic potential of PF11. This review is designed to create a synthesis of the PF11 literature in a methodological sense; a quantitative analysis of the PK data, and the paradox of bioavailability and efficacy; a mechanism model with the clear caveat that certain elements are speculative and require direct verification; a quantitative evaluation of translational challenges; and a road map for future directions based on defined milestones. Through the use of such a critical approach, it is hoped that the review will move beyond a simple summary of the literature.

### 1.1 Literature search methodology

#### 1.1.1 Search strategy

A systematic literature search was conducted in accordance with the PRISMA (Preferred Reporting Items for Systematic Reviews and Meta-Analyses) guidelines. Four electronic databases were searched from inception through 31 December 2024: PubMed/MEDLINE, Web of Science, Embase, and the China National Knowledge Infrastructure (CNKI). The following search strings were used:

PubMed/MEDLINE: (“pseudoginsenoside F11” OR “pseudoginsenoside-F11” OR “PF11” OR “20(S)-pseudoginsenoside F11” OR “pseudo-ginsenoside F11”) AND (pharmacology OR pharmacokinetic OR mechanism OR neuroprotection OR anti-inflammation OR antitumor OR cardio protection OR antioxidant).

Web of Science: Same terms with Boolean operators adapted to platform syntax.

Embase: Same core terms with Embase-specific MeSH equivalents. CNKI: Equivalent Chinese-language terms (“伪人参皂苷F11” OR “拟人参皂苷F11”).

#### 1.1.2 Inclusion and exclusion criteria

Studies were included if they: (1) investigated PF11 as a primary compound of interest, not merely as part of a crude extract; (2) reported original experimental data (*in vitro*, *in vivo*, or *ex vivo*); (3) provided sufficient methodological detail for critical appraisal; and (4) were published in peer-reviewed journals in English or Chinese.

Studies were excluded if they: (1) investigated crude ginseng extracts without PF11-specific data; (2) were conference abstracts, editorials, or narrative commentaries without original data; (3) were duplicates (identified by DOI or PMID cross-referencing); or (4) could not be retrieved in full text after two independent attempts.

#### 1.1.3 Screening workflow and final corpus

Initial database searches yielded 347 records. After automated deduplication, 241 unique records remained. Title and abstract screening by two independent reviewers (consensus required for inclusion/exclusion disagreements; Cohen’s κ = 0.82) excluded 118 records. Full-text review of the remaining 123 studies resulted in the exclusion of a further 44 records due to failure to meet inclusion criteria. The final corpus comprised 79 studies, which form the evidentiary basis of this review.

It is important to acknowledge a potential publication bias in this literature: positive results are significantly more likely to be published than null findings, and the majority of included studies (n = 71, 80%) reported beneficial effects of PF11. This limitation is explicitly carried forward as a caveat in the interpretation of the pharmacological evidence throughout this review.

## 2 Phytochemical profile

### 2.1 Molecular structure

PF11 is a member of the ocotillol-type ginsenosides, a specialized subclass of dammarane-type triterpenoid saponins defined by a tetrahydrofuran ring at the C-20 position in addition to the canonical dammarane skeleton. Its molecular formula is C_42_H_72_O_14_, corresponding to a molecular weight of 801.01 g/mol (Liu J. et al., 2017). PF11’s aglycone moiety is ocotillol, specifically (20S, 24R)-dammarane-20,24-epoxy-3β,6α,12β,25-tetraol (Han et al., 2020). This moiety features a tetracyclic dammarane skeleton consisting of four trans-fused rings, labeled A, B, C, and D. PF11’s most distinctive structural feature is a tetrahydrofuran moiety formed by an epoxide bridge between the C-20 and C-24 positions, along with a tertiary hydroxyl group at C-25 substituted with two methyl groups. This ocotillol-type configuration sets PF11 apart from the more prevalent protopanaxadiol and protopanaxatriol ginsenosides abundant in Asian ginseng (Christensen, 2009).

**FIGURE 1:**
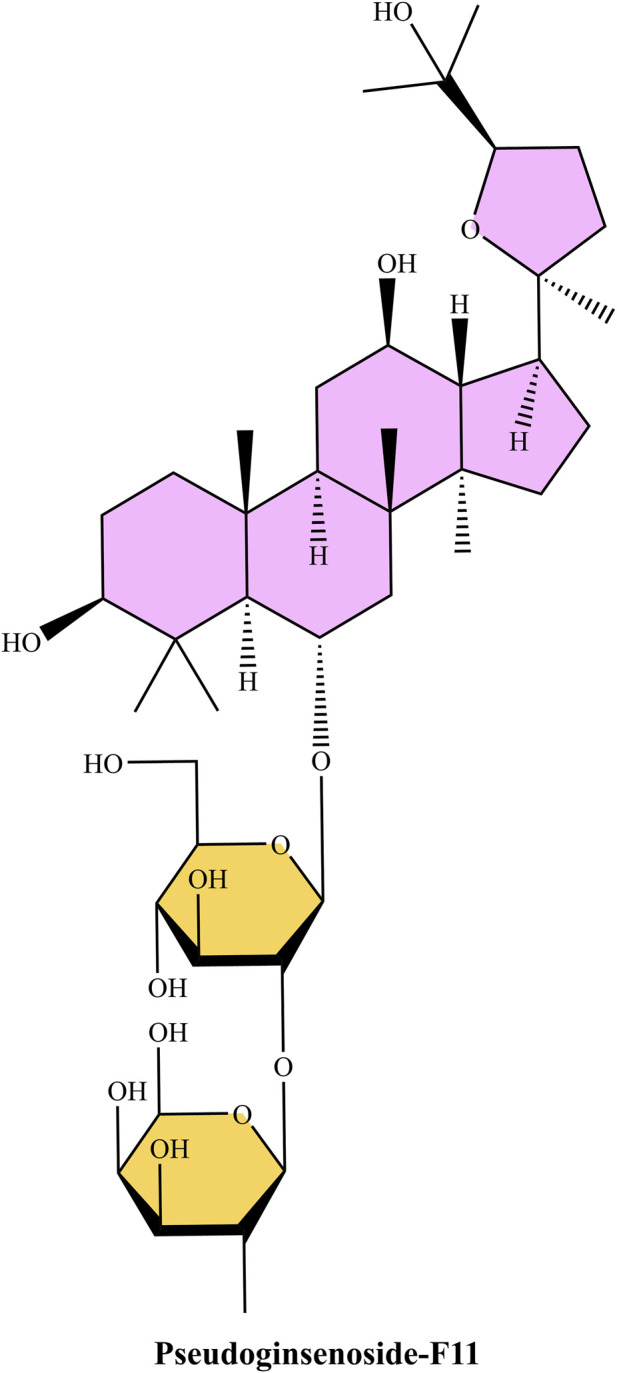
Chemical structure of PF11.

The stereochemistry at the C-20 and C-24 positions is particularly important, with PF11 exhibiting the 20(S),24(R) configuration. This specific stereoisomeric arrangement is crucial to its biological activities and differentiates it from other ocotillol-type derivatives such as 24(S)-pseudoginsenoside F11, which possesses distinct pharmacological profiles (Wang et al., 2012) (Figure 1).

### 2.2 Physicochemical properties

Purified PF11 presents as a white to off-white amorphous powder. Consistent with its saponin nature, it exhibits typical solubility properties: it is soluble in polar solvents such as water, methanol, ethanol, and dimethyl sulfoxide (DMSO) (Li et al., 2016).

High-resolution mass spectrometry confirms PF11’s accurate mass as 800.4922 Da. Under electrospray ionization mass spectrometry (ESI-MS), PF11 typically forms a [M + Na] + adduct ion at m/z 823 and a [M-H]- ion at m/z 799 in negative ion mode. It also yields characteristic fragment ions through sequential loss of sugar moieties and dehydration of the aglycone (Chan et al., 2000).

PF11 lacks prominent UV chromophores, posing challenges for detection via conventional HPLC-UV methods. This is due to the absence of aromatic rings or extensive conjugated systems in its structure. Thus, detection commonly relies on evaporative light scattering detection, charged aerosol detection, or mass spectrometry. These techniques provide enhanced sensitivity for non-UV-absorbing compounds (Sun et al., 2012).

PF11’s chemical structure and physicochemical properties embody a complex natural product architecture, featuring a dammarane skeleton, a unique tetrahydrofuran ring system, specific stereochemical configurations, and a disaccharide substitution pattern. These structural features collectively determine the compound’s solubility profile, spectroscopic characteristics, stability, and biological activities. Understanding these fundamental chemical properties is essential for developing analytical methods, quality control protocols, and structure-activity relationship studies that seek to exploit the therapeutic potential of this important natural product.

## 3 Pharmacological activities

### 3.1 Neurological disorders

#### 3.1.1 Ischemic stroke

PF11 exhibits significant neuroprotective potential in various rodent models of cerebral ischemia, covering both transient and permanent occlusion models. It consistently reduces infarct volume, cerebral edema, and sensorimotor impairments, with protective effects remaining evident even when administered after ischemia onset. For instance, in rats subjected to embolic middle cerebral artery occlusion (eMCAO), intravenous PF11 (4–36 mg/kg) administered daily for 3 days prior to occlusion significantly decreased infarct size by up to 25%. It also reduced brain water content and improved neurological scores at 24 h post-occlusion, as evaluated by 2,3,5-triphenyltetrazolium chloride (TTC) staining and behavioral assessments (Gao et al., 2021). Similarly, in mice with transient middle cerebral artery occlusion (tMCAO), oral PF11 (16–32 mg/kg) given daily from 1 week before to 6 weeks after ischemia not only lowered mortality from 40% to approximately 20% but also enhanced long-term outcomes. These include rotarod performance and grip strength improvements lasting up to 42 days post-stroke (Yuan et al., 2020). These findings underscore PF11’s broad therapeutic window, spanning acute neuroprotection to chronic recovery, which may overcome the limitations of conventional agents like edaravone that often have narrower efficacy timelines.

The mechanisms underlying PF11’s neuroprotective effects are diverse, with calcium homeostasis modulation being prominent to counteract excitotoxic cascades triggered by ischemia. In tMCAO rats, a single intravenous dose of PF11 administered at the onset of reperfusion suppressed μ-calpain autolysis and preserved sarcoplasmic/endoplasmic reticulum Ca^2+^-ATPase 2 levels. This alleviated endoplasmic reticulum stress markers such as C/EBP homologous protein (CHOP) and reduced neuronal apoptosis in the cortex at 24 h (Zhang et al., 2019a). This calcium-stabilizing effect was replicated *in vitro*: pretreatment with PF11 (30–100 μM) restored ATP content, reduced intracellular Ca^2+^ concentrations, and normalized N-methyl-D-aspartate receptor subunits (NR1, NR2A, NR2B) along with postsynaptic density protein 95 and neuronal nitric oxide synthase in primary cortical neurons challenged by oxygen-glucose deprivation/reoxygenation (OGD/R) (Zhang et al., 2019a). Such interventions likely interrupt the vicious cycle of Ca^2+^ influx and protease activation, preserving synaptic structures and preventing delayed cell death. Importantly, these effects explain PF11’s sustained benefits in mitigating gait asymmetries and propulsion deficits for up to 2 weeks post-tMCAO, establishing a mechanistic link between acute ionic dysregulation and chronic functional recovery.

PF11 further counteracts ischemic injury by reversing impairments in the autophagy-lysosomal pathway (ALP), a critical clearance system disrupted during stroke that leads to toxic aggregate accumulation. In rats with permanent MCAO (pMCAO), PF11 (6–12 mg/kg, i. v.) administered 0.5 h post-occlusion reduced autophagosome buildup, as indicated by decreased LC3-II and SQSTM1/p62 levels. It also enhanced lysosomal markers such as cathepsin D and lysosomal-associated membrane protein 1 in the cortex at 24 h (Fu et al., 2021). This restoration involved calcineurin-dependent nuclear translocation of transcription factor EB (TFEB), a master regulator of ALP genes. Cyclosporine A blocked PF11’s effects on TFEB localization and ALP flux in OGD-treated neurons (Fu et al., 2021). Earlier findings in pMCAO models confirmed this: PF11 (3–12 mg/kg) improved lysosome-autophagosome fusion and reduced ubiquitin aggregates, effects abolished by chloroquine, a lysosomal inhibitor (Liu Y. Y. et al., 2017). By promoting degradative processes, PF11 prevents secondary neurodegeneration caused by undigested debris, a key factor in prolonged ischemic pathology. This ALP-enhancing property correlates with PF11’s dose-dependent reduction in TUNEL-positive apoptotic cells and glial activation, indicating a comprehensive cellular cleanup that supports neuronal viability beyond immediate insult resolution.

Beyond cellular clearance, PF11 modulates neuroinflammatory responses by regulating microglial/macrophage phenotypes and phagocytic functions, creating a reparative brain microenvironment. In tMCAO rats, oral PF11 (12 mg/kg) advanced the peak of anti-inflammatory M2 microglia/macrophages from day 14 to day 3 post-ischemia in the ipsilateral striatum and cortex. This was accompanied by increased interleukin-10 and decreased interleukin-1β mRNA levels (Hou et al., 2021). This phenotypic shift depended on Jumonji domain-containing protein 3 (Jmjd3), as PF11 upregulated M2 marker expression in OGD/R-conditioned microglia—an effect reversed by the Jmjd3 inhibitor GSK-J4. Complementarily, in pMCAO rats, PF11 (12 mg/kg, i. v.) accelerated myelin debris clearance via complement receptor 3 (CR3/CD11b), enhancing phagocytic uptake in microglia at 24 h. Anti-CD11b antibodies eliminated PF11’s effects on reducing neuronal loss and edema (Liu et al., 2020). These immunomodulatory actions likely synergize with PF11’s other pathways, dampening pro-inflammatory amplification while facilitating debris clearance—critical for axonal regeneration and functional recovery. The specificity of CR3 involvement, without affecting non-specific phagocytosis such as latex bead uptake, highlights PF11’s targeted enhancement of stroke-relevant clearance mechanisms.

PF11’s vascular protective effects extend to thromboembolic scenarios, where it mitigates thromboinflammation and preserves the blood-brain barrier (BBB). In eMCAO rats, pretreatment with PF11 (4–36 mg/kg) reduced microvascular thrombi, fibrin deposition, and leukocyte infiltration in the ischemic cortex at 24 h. It also downregulated platelet glycoproteins Ibα and VI, as well as contact-kinin pathway components like bradykinin (Gao et al., 2021). This preserved tight junction proteins (claudin-5, occludin, ZO-1) and reduced matrix metalloproteinase-9 expression, minimizing Evans blue extravasation and ultrastructural BBB disruptions observed via transmission electron microscopy. By inhibiting these vascular-inflammatory interactions, PF11 reduces edema and secondary hemorrhage risks, complementing its neuronal protective effects in embolic strokes. Such multifaceted vascular stabilization may expand PF11’s applicability in heterogeneous clinical stroke presentations.

Emerging evidence links PF11’s efficacy to NMDAR subunit-specific signaling, particularly NR2A-mediated pro-survival pathways. In pMCAO rats, PF11 (3–12 mg/kg, i. v.) inhibited calpain 1 activation and α-fodrin breakdown, while upregulating Ca^2+^/calmodulin-dependent protein kinase II-α at 24 h. This ultimately enhanced NR2A expression and AKT-CREB phosphorylation in the cortex and striatum (Liu et al., 2022). The NR2A antagonist NVP-AAM077 abrogated these effects, including PF11’s improvements in neuronal viability and CREB activation in OGD neurons, confirming pathway dependency. This NR2A-centric mechanism aligns with PF11’s promotion of neurogenesis. In tMCAO mice, it increased brain-derived neurotrophic factor and doublecortin in neurogenic niches, facilitating neuroblast migration and newborn neuron survival for up to 42 days (Yuan et al., 2020). Collectively, these pathways demonstrate PF11’s integrated protective strategy: stabilizing ion fluxes, clearing debris, suppressing inflammation, and activating regenerative signals to counteract ischemia’s multifactorial damage.

Despite these compelling findings, gaps remain in translating PF11’s benefits to clinical practice. Optimal dosing and timing suggest pharmacokinetic refinements could widen its therapeutic window. Moreover, PF11 occasionally exhibits a bell-shaped dose-response—higher doses lose efficacy in reducing infarct size but retain behavioral benefits. This phenomenon warrants mechanistic investigation, potentially involving off-target effects. Nevertheless, PF11’s multi-target profile suggests potential complementarity with existing agents such as thrombolytics, though direct comparative or combination studies have yet to be conducted in humans. Future studies should explore its human pharmacokinetics and safety to advance clinical translation.

#### 3.1.2 Alzheimer’s disease

Early research into its cognitive-enhancing effects focused on scopolamine-induced memory impairment models. Scopolamine, a muscarinic antagonist that disrupts cholinergic signaling, mimics key features of AD-related amnesia (Chen and Yeong, 2020). In a seminal study, intragastric PF11 administration at 2 or 4 mg/kg for 5 days significantly prolonged the shortened latency in passive avoidance tasks induced by scopolamine (2 mg/kg, i. p.) in mice, without altering baseline behavior in naive animals (Li et al., 1999). This antagonistic effect extended to active avoidance paradigms in rats, where PF11 (1.2 or 2.4 mg/kg) alleviated scopolamine-induced deficits in avoidance percentage and latency. These results suggest PF11 counteracts cholinergic dysfunction and lay the groundwork for exploring its broader therapeutic potential, indicating it may facilitate memory consolidation and retrieval through mechanisms beyond simple anticholinergic reversal.

Building on these findings, subsequent studies explored PF11’s efficacy in more direct AD models, such as those involving amyloid-beta (Aβ) oligomers—central drivers of AD pathogenesis due to their neurotoxicity. In mice receiving intracerebroventricular injection of oligomeric Aβ1-42, oral PF11 at 1.6 or 8 mg/kg for 15 days improved cognitive performance in the Morris water maze and step-through tests. It also reduced Aβ accumulation in the cortex and hippocampus (Wang et al., 2013a). Notably, PF11 restored activities of antioxidant enzymes (superoxide dismutase [SOD], glutathione peroxidase [GSH-Px]), lowered malondialdehyde (MDA) levels, and downregulated pro-apoptotic markers (JNK2, p53, cleaved caspase-3) in the hippocampus. This indicates PF11’s anti-amnesic effects in Aβ-induced models stem from combating oxidative stress and neuronal apoptosis—pathologies exacerbated by Aβ oligomers. Its regulatory effect on amyloid precursor protein (APP) expression further supports interference with amyloidogenesis, potentially by shifting APP processing away from the amyloidogenic pathway.

In transgenic models recapitulating familial AD, PF11 exerted robust neuroprotective effects. For example, a 4-week regimen of PF11 (8 mg/kg) significantly improved performance in novel object recognition and Morris water maze tasks in APP/PS1 mice. This was accompanied by reduced APP and Aβ1-40 levels in cortical and hippocampal regions (Wang et al., 2013a). These benefits were linked to enhanced antioxidant defenses and attenuated histopathological alterations, highlighting PF11’s ability to target core AD hallmarks like plaque formation and synaptic loss. Extending to sporadic AD models, PF11 alleviated cognitive impairments in senescence-accelerated mouse prone 8 mice—animals that exhibit age-related Aβ deposition and tau hyperphosphorylation. Oral PF11 (up to 8 mg/kg) administered for 3 months starting at 6 months of age reversed memory deficits in novel object recognition and spatial navigation tests. It also promoted APP transport from the cytoplasm to the plasma membrane, normalized BACE1 expression, and modulated PP2A methylation via increased LCMT-1 levels—thereby reducing tau hyperphosphorylation at sites such as Ser396 and Thr231 (Zhang et al., 2019c). These actions underscore PF11’s multifaceted regulation of protein phosphatases, which may prevent neurofibrillary tangle formation and synaptic dysfunction in aging brains.

Further insights came from models of mild cognitive impairment and sporadic AD induced by metabolic stressors. In D-galactose-treated mice, PF11 (2–16 mg/kg for 9 weeks) mitigated hippocampal neuronal loss and microglial activation. It also suppressed the NLRP3 inflammasome by reducing AGEs/RAGE signaling and bolstered Nrf2/GST pathways to enhance SOD activity and GSH levels, countering MDA accumulation (Zhang et al., 2019b). Similarly, in streptozotocin (STZ)-infused rats, PF11 (2–8 mg/kg for 4 weeks) dose-dependently improved learning and memory in nest-building, Y-maze, and Morris water maze assays. It also protected synaptic integrity and reduced tau phosphorylation by restoring insulin signaling and inhibiting the calpain I/CDK5 pathway (Zhu et al., 2021). At higher doses, PF11 exhibited efficacy comparable to donepezil, suggesting it targets upstream mechanisms like insulin desensitization—an increasingly recognized factor in AD etiology.

PF11’s therapeutic scope extends to vascular dementia, a condition often overlapping with AD pathologies. In rats with chronic cerebral hypoperfusion induced by two-vessel occlusion, PF11 (6–24 mg/kg for 4 weeks) alleviated white matter injury and cognitive decline by enhancing ALP function in oligodendrocytes. This was evidenced by increased autophagosome formation, upregulated mTOR-mediated autophagy markers (e.g., ULK1, BECN1), and elevated lysosomal proteins (LAMP1/2, CTSD) (Wang et al., 2024a). It also reduced aberrant SQSTM1 accumulation and glial activation, preserving myelin integrity. This ALP modulation aligns with findings in microglial models, where PF11 reversed oligomeric Aβ-induced endosome-lysosome defects by promoting TFEB nuclear translocation, Rab5-to-Rab7 conversion, and lysosomal enzyme expression—thus facilitating Aβ clearance (Yao et al., 2019). Collectively, these studies demonstrate PF11’s ability to restore degradative pathways impaired in AD, potentially by stabilizing lysosomal pH and enhancing endosomal maturation.

Accumulated evidence positions PF11 as a promising multi-target agent for AD, addressing amyloidosis, tauopathy, oxidative stress, inflammation, and ALP dysfunction across diverse models. However, despite compelling preclinical data, a translational gap persists. Dose-response inconsistencies across studies demand pharmacokinetic optimization for human relevance. Future research should elucidate PF11’s blood-brain barrier penetration and long-term safety, alongside clinical trials to validate efficacy in AD patients. Overall, PF11’s profile suggests it could complement existing therapies, highlighting the value of natural saponins in neurodegenerative drug discovery.

#### 3.1.3 Parkinson’s disease

PD is a progressive neurodegenerative disorder characterized by the loss of dopaminergic neurons in the substantia nigra and subsequent motor deficits (Liu et al., 2025). Research confirms PF11’s ability to mitigate oxidative stress, neuroinflammation, and neuronal damage in experimental PD models, with therapeutic potential mediated through multifaceted mechanisms. Notably, studies show PF11 can restore behavioral impairments and biochemical markers of dopaminergic dysfunction, in preclinical settings, warranting further investigation to evaluate its clinical potential.

In a rat PD model induced by 6-hydroxydopamine (6-OHDA), PF11 exhibited significant neuroprotective effects. Oral PF11 (3–12 mg/kg) administered for 3 weeks before and after unilateral medial forebrain bundle lesioning markedly improved locomotor activity, motor balance, coordination, and reduced apomorphine-induced rotations. These behavioral improvements were accompanied by increased tyrosine hydroxylase expression in the substantia nigra and elevated extracellular dopamine levels in the striatum, indicating preserved dopaminergic integrity. Furthermore, PF11 reduced hydroxyl radical formation and enhanced ascorbic acid release, underscoring antioxidant properties as a key driver of its neuroprotection (Wang et al., 2013b). This study provides compelling evidence that PF11 not only alleviates symptomatic manifestations but also targets underlying oxidative imbalances central to PD pathogenesis.

Beyond direct neuronal protection, PF11’s anti-inflammatory actions contribute to its efficacy against PD-related neurodegeneration. In lipopolysaccharide (LPS)-activated microglial cells, PF11 suppressed reactive oxygen species production and reduced levels of proinflammatory mediators, including nitric oxide, prostaglandin E2, interleukin-1β, interleukin-6, and tumor necrosis factor-α. This inhibition was achieved by disrupting the interaction between Toll-like receptor 4 (TLR4) and myeloid differentiation primary response 88 (MyD88), thereby attenuating downstream signaling via transforming growth factor-β-activated kinase 1 (TAK1), IκB kinase (IKK), nuclear factor-κB (NF-κB), mitogen-activated protein kinases (MAPKs), and Akt pathways. *In vivo* validation in LPS-injected mice confirmed reduced microglial activation and proinflammatory factor expression in the cortex and hippocampus following PF11 treatment (8 mg/kg) (Wang et al., 2014). These findings illustrate PF11’s capacity to curb microglia-mediated neurotoxicity, which exacerbates dopaminergic cell death in PD, linking its anti-inflammatory and neuroprotective profiles.

Comparative analyses of ginseng-derived compounds contextualize PF11’s role in PD management. A comprehensive review of ginseng’s neuroprotective activities highlights PF11 as a minor ginsenoside with notable anti-PD potential, alongside major components like Rb1 and Rg1. PF11’s unique ocotillol structure may confer advantages in modulating N-methyl-D-aspartate receptor activity, reducing nigral iron accumulation, and inhibiting neurotoxin-induced apoptosis—mechanisms consistent with those observed in experimental PD models (González-Burgos et al., 2015). While PF11 shares antioxidative and anti-apoptotic traits with other ginsenosides, its specificity in targeting TLR4-mediated inflammation distinguishes it, potentially making it more suitable for inflammation-dominant PD subtypes.

**FIGURE 2:**
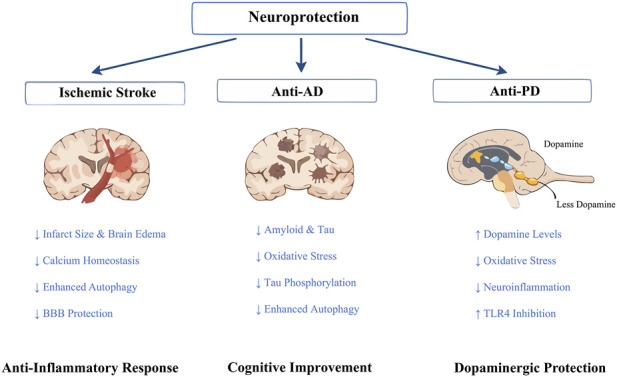
Neuroprotective effects of PF11 in ischemic stroke, AD, and PD. Note: This schematic illustrates the multifaceted neuroprotective mechanisms of PF11, including reducing infarct size and brain edema in ischemic stroke, inhibiting amyloid-β aggregation and tau hyperphosphorylation in AD, and restoring dopamine levels while mitigating oxidative stress in PD, ultimately exerting anti-inflammatory and cognitive-improving effects.

Despite these promising outcomes, several aspects require critical evaluation. Studies predominantly rely on rodent models, which may not fully recapitulate human PD etiology, including genetic factors like α-synuclein aggregation. Dose-dependent effects in preclinical settings indicate a therapeutic window, but pharmacokinetic data on PF11’s bioavailability and brain penetration remain limited, raising questions about translational feasibility. Overall, PF11 represents a compelling natural compound for PD neuroprotection, with its dual antioxidant and anti-inflammatory mechanisms providing a strong rationale for further phytomedicine development (Figure 2).

### 3.2 Effects on drug tolerance

Tolerance, characterized by diminished responsiveness to a drug following repeated exposure, poses a significant challenge in managing substance abuse disorders. Research indicates that PF11 interferes with the adaptive neurochemical changes underlying tolerance, offering insights into its therapeutic utility as an adjunct in addiction treatment.

Initial investigations into PF11’s effects on morphine tolerance revealed its capacity to prevent the development of analgesic tolerance in rodent models. In mice subjected to chronic morphine administration (10 mg/kg subcutaneously daily for 9 days), PF11 at oral doses of 4 and 8 mg/kg significantly preserved the analgesic response to a challenge dose of morphine (5 mg/kg) in the tail-pinch test, as evidenced by sustained biting latencies compared to morphine-alone groups (Li et al., 2000). This antagonism suggests that PF11 may disrupt the cellular adaptations, such as receptor desensitization or downstream signaling alterations, that contribute to tolerance buildup. Notably, the effect was dose-dependent, with higher doses yielding more pronounced inhibition, highlighting PF11’s targeted interference without altering baseline analgesia.

Beyond direct tolerance to analgesia, PF11 has shown efficacy in countering reverse tolerance, or behavioral sensitization, which manifests as heightened locomotor responses to repeated drug exposure and is implicated in craving and relapse. In studies involving morphine-induced sensitization, oral PF11 (4 and 8 mg/kg) administered prior to daily morphine injections (10 mg/kg subcutaneously for 7 days) markedly attenuated the progressive increase in locomotor activity observed on day 7 compared to controls (Li et al., 2000). Complementary findings extended this to neurochemical levels, where repeated PF11 co-administration normalized extracellular glutamate reductions in the medial prefrontal cortex (mPFC) induced by chronic morphine, a change linked to sensitization processes (Hao et al., 2007). These results imply that PF11’s modulation of glutamatergic transmission could underpin its anti-sensitization properties, potentially by stabilizing excitatory pathways that exacerbate addictive behaviors.

The anti-tolerance profile of PF11 extends to stimulants like methamphetamine (METH), where it similarly curbs sensitization. Chronic METH exposure (1 mg/kg subcutaneously for 6 days) elicited robust locomotor sensitization in mice, but co-treatment with PF11 (1, 4, or 8 mg/kg orally) dose-dependently reduced this effect, with higher doses approaching baseline activity levels (Fu et al., 2016). Micro dialysis analyses further elucidated that PF11 mitigated METH-induced elevations in extracellular dopamine in the nucleus accumbens while enhancing gamma-aminobutyric acid (GABA) release, suggesting a dual regulation of dopaminergic and GABAergic neurons to prevent hyper-responsiveness (Fu et al., 2016). This GABAergic enhancement may serve as a compensatory mechanism to dampen METH’s reinforcing effects, aligning with PF11’s broader neuroprotective role.

Mechanistically, PF11’s influence on tolerance appears tied to its interaction with opioid receptors and intracellular signaling. In Chinese hamster ovary cells expressing μ-opioid receptors, PF11 competitively inhibited morphine binding and reversed morphine’s suppression of cyclic AMP production, indicating direct antagonism at the receptor level (Li et al., 2001). Such actions could prevent the desensitization and internalization of μ-receptors that drive tolerance. However, while these cellular findings provide a foundation, *in vivo* studies suggest additional involvement of glutamatergic and GABAergic systems, warranting further exploration of PF11’s multi-target pharmacology.

**FIGURE 3:**
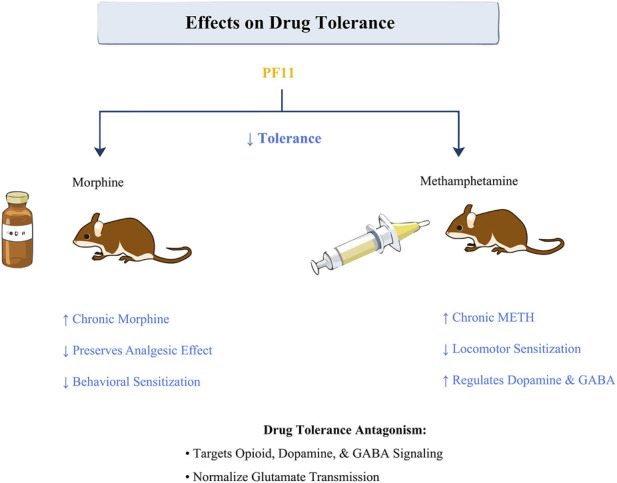
PF11 attenuates drug tolerance to morphine and methamphetamine. Note: This diagram demonstrates PF11’s ability to antagonize drug tolerance by preserving the analgesic effect of chronic morphine, reducing locomotor sensitization to methamphetamine, and modulating opioid, dopamine, GABA, and glutamate signaling pathways.

Overall, the evidence positions PF11 may attenuate tolerance development across morphine and methamphetamine models, with consistent antagonism observed in rodent studies. These findings provide a mechanistic rationale for further investigation; however, no clinical data exist to confirm these effects in humans. Translating preclinical dose regimens and elucidating long-term safety remain critical unresolved challenges. Future research should evaluate PF11’s efficacy in well-designed human studies before its potential role in addiction pharmacotherapy can be adequately assessed (Figure 3).

### 3.3 Myocardial protection

Early studies on its derivatives laid the groundwork for understanding its role in cardiac health, revealing mechanisms involving antioxidant enhancement and modulation of adrenergic signaling pathways. A foundational study explored the cardioprotective effects of ocotillol—a metabolite of PF11 — in a rat model of isoproterenol-induced myocardial injury. Isoproterenol, a β-adrenergic agonist, induces ischemic stress via oxidative damage and necrosis (Yu et al., 2007). Oral ocotillol administration at 5–20 mg/kg for 8 days significantly attenuated elevations in lactate dehydrogenase (LDH), a key marker of cardiac tissue damage. It also restored SOD activity and reduced MDA content in heart tissues. Histopathological assessments confirmed ocotillol alleviated necrotic lesions and preserved myocardial architecture. These results indicate ocotillol’s protection stems from enhancing cardiac antioxidant defenses to counter free radical surges triggered by isoproterenol. Given ocotillol forms via gastrointestinal metabolism or chemical degradation of PF11, the precursor PF11 may exert similar *in vivo* benefits.

Direct evidence of PF11’s anti-ischemic efficacy came from rat coronary artery ligation experiments, which mimic acute myocardial infarction by restricting blood flow and inducing hypoxic damage (Zhang et al., 2017). Intragastric PF11 pretreatment at 6 and 12 mg/kg for 7 days prior to ligation markedly improved cardiac function, as shown by enhanced left ventricular ejection fraction and fractional shortening via echocardiography. Biochemically, PF11 reduced serum levels of creatine kinase, LDH, aspartate transaminase, MDA, and cardiac troponin T, while increasing SOD activity—indicators of reduced oxidative stress and cellular leakage. Infarct size was significantly diminished, and flow cytometry combined with Western blotting revealed downregulated β1-adrenoceptor expression in cardiac tissues. Molecular docking simulations supported potential binding interactions between PF11 and β1-AR, suggesting a receptor-antagonistic mechanism that curbs excessive adrenergic stimulation, a known driver of ischemic remodeling. These findings extend beyond symptom relief, positioning PF11 as a modulator of pathological ischemia-related signaling those better preserves ventricular integrity than lower doses or controls.

Further validation of PF11’s cardioprotective profile came from integrated metabolomics and zebrafish screening, which identified it as a pharmacodynamic marker against heart failure (Dong et al., 2022). In verapamil-induced heart failure models using AB-line zebrafish larvae, PF11 was among differential metabolites in American ginseng extracts with varying efficacy across production regions. Network pharmacology predictions and quantitative real-time PCR confirmed PF11 regulates genes linked to cardiac energy metabolism and anti-apoptotic pathways, such as those involved in AMPK activation. This high-throughput model demonstrated PF11’s ability to restore heart rate and ventricular function, aligning with mammalian data while providing a more efficient platform for mechanistic insights. Consistency across models highlights PF11’s broad applicability, though efficacy variations may stem from bioavailability differences or synergistic interactions with other ginsenosides.

**FIGURE 4:**
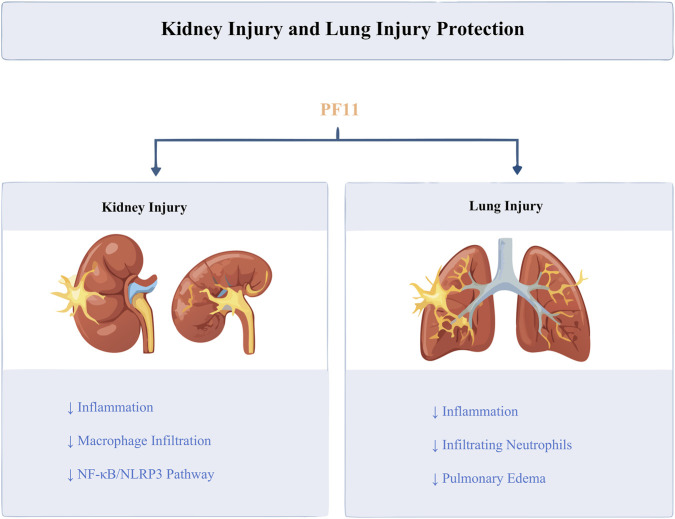
Protective effects of PF11 against acute kidney injury and acute lung injury. Note: This diagram depicts PF11’s protective actions in AKI and ALI, primarily through suppressing inflammation, macrophage/neutrophil infiltration, and the NF-κB/NLRP3 signaling pathway, thereby reducing tissue damage and pulmonary edema.

Collectively, these studies establish PF11 as a promising myocardial protective agent, acting through multifaceted mechanisms including antioxidation, β1-AR inhibition, and metabolic modulation. However, challenges remain, such as clarifying dose-dependent pharmacokinetics and long-term clinical safety. Future research may explore synergies with conventional β-blockers or antioxidants to enhance therapeutic indices (Figure 4).

### 3.4 Kidney injury and lung injury protection

Research shows PF11 regulates inflammatory responses and cellular signaling pathways to exert protection without adverse effects on normal tissues. In LPS-induced acute kidney injury (AKI), PF11 exerts significant nephroprotective effects by modulating macrophage polarization and downstream signaling cascades. Specifically, it reduces renal histopathological alterations, lowers inflammatory cytokine levels, and diminishes macrophage infiltration in affected tissues. This protection is mediated by suppressing the NF-κB/NOD-like receptor pyrin domain-containing 3 (NLRP3)/interleukin-1β (IL-1β) pathway, which amplifies inflammation during AKI (Wang et al., 2024b). Notably, macrophage depletion with clodronate liposomes confirmed PF11’s benefits depend largely on immune cell regulation, as macrophage absence abolished its ability to alleviate kidney damage and inhibit the pathway. These findings underscore PF11’s role in reshaping the inflammatory microenvironment by promoting a shift from pro-inflammatory M1 macrophages to anti-inflammatory M2c subtypes, facilitating tissue repair and limiting excessive immune activation.

PF11’s protective mechanisms extend to cisplatin-induced nephrotoxicity, a common chemotherapy complication driven by oxidative stress and renal tubular cell apoptosis. PF11 pretreatment significantly attenuates elevations in blood urea nitrogen and creatinine—markers of renal dysfunction—while ameliorating histopathological changes such as tubular degeneration and vacuolization. At the molecular level, it suppresses p53 activation, reverses the pro-apoptotic Bax/Bcl-2 ratio, and enhances antioxidant defenses by increasing glutathione peroxidase and SOD activities while reducing lipid peroxidation (Wang et al., 2014). These actions collectively inhibit tubular cell apoptosis, as evidenced by decreased TUNEL-positive cells and cleaved caspases. Importantly, this nephroprotection does not compromise cisplatin’s antitumor efficacy. In fact, PF11 enhances cisplatin’s efficacy in murine melanoma and lung cancer xenograft models, suggesting synergistic potential to improve oncology therapeutic outcomes. PF11’s antioxidant properties align with cisplatin toxicity pathophysiology, where reactive oxygen species accumulation drives cell death, indicating it acts as a targeted scavenger to preserve renal integrity without interfering with cisplatin’s DNA-damaging effects on tumor cells.

PF11 also provides robust protection against LPS-induced acute lung injury (ALI), characterized by neutrophil-dominated inflammation and edema. It alleviates lung histopathological damage, reduces the wet/dry weight ratio, and lowers protein concentrations and inflammatory cell counts in bronchoalveolar lavage fluid. The compound suppresses expression of pro-inflammatory cytokines (IL-6, TNF-α, IL-1β) and decreases myeloperoxidase activity—an indicator of neutrophil accumulation (Wang et al., 2019). Mechanistically, PF11 inhibits neutrophil infiltration by downregulating macrophage inflammatory protein-2 and intercellular adhesion molecule-1, while accelerating neutrophil clearance via enhanced apoptosis and phagocytosis by alveolar macrophages. This dual action—curbing influx and promoting resolution—highlights PF11’s ability to restore lung homeostasis, potentially through NF-κB signaling interference as observed in related studies. Consistency across models points to a shared anti-inflammatory theme, where PF11 modulates innate immune responses to prevent cascading tissue injury.

**FIGURE 5:**
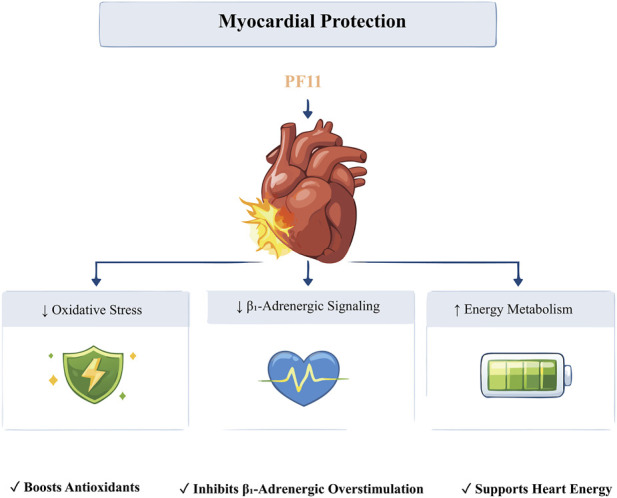
Myocardial protective mechanisms of PF11. Note: This schematic outlines PF11’s cardioprotective effects, which include attenuating oxidative stress, inhibiting excessive β_1_-adrenergic signaling, and enhancing myocardial energy metabolism, collectively boosting antioxidant defenses and supporting cardiac function.

Collectively, these studies demonstrate PF11’s versatility in protecting against organ-specific injuries induced by bacterial endotoxins or chemotherapeutic agents. However, reliance on animal models limits direct extrapolation to humans. Further investigations into dose-response relationships, long-term safety, and interactions with pathways like TLR4 or Nrf2 could refine its clinical potential (Figure 5).

### 3.5 Tissue regeneration and metabolic regulation

Random-pattern skin flaps are widely used in reconstructive surgery, but distal flap necrosis remains a major clinical challenge that limits flap size and reliability. Autophagy activation has emerged as a potential strategy to enhance flap survival by promoting cellular adaptation to ischemia-reperfusion injury. Zhou et al. investigated whether PF11 improves random-pattern skin flap viability via autophagy induction. In a rat skin flap model, PF11 administration significantly enhanced flap survival, increased blood flow, reduced tissue edema, and improved histological appearance (Zhou et al., 2021). Mechanistic analyses revealed PF11 promotes autophagy through activating the AMPK-mTOR-TFEB signaling axis. Specifically, PF11 activates AMPK, which in turn inhibits mechanistic target of rapamycin (mTOR) and induces TFEB nuclear translocation. As a master regulator of autophagy and lysosome biogenesis, TFEB activation enhances autophagic flux and improves cellular stress adaptation. These findings suggest PF11 may support tissue regeneration and wound healing through autophagy-mediated cytoprotective mechanisms.

**FIGURE 6:**
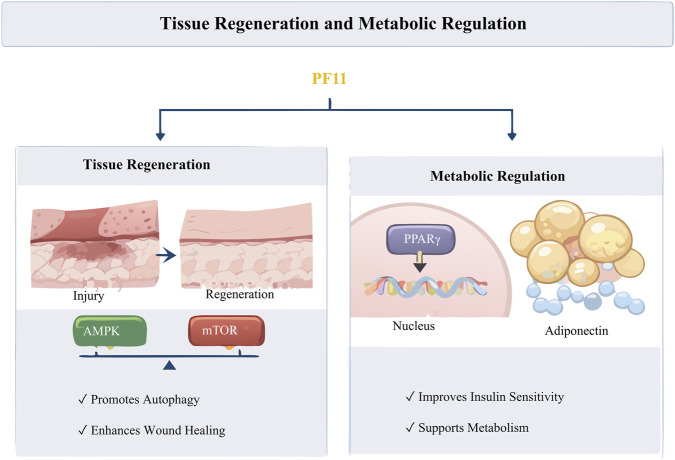
PF11 promotes tissue regeneration and metabolic regulation. Note: This schematic illustrates PF11’s dual roles in enhancing tissue regeneration (via AMPK/mTOR-mediated autophagy and wound healing) and regulating metabolism (via PPARγ activation and adiponectin modulation), which improves insulin sensitivity and reduces inflammation.

PPARγ is a nuclear hormone receptor that acts as a master regulator of adipocyte differentiation, lipid metabolism, and glucose homeostasis. Wu et al. (2013) identified PF11 as a novel partial PPARγ agonist through screening natural compounds for adipogenic activity. In 3T3-L1 preadipocytes, PF11 promoted differentiation and lipid accumulation—albeit to a lesser extent than the full agonist rosiglitazone—confirming its partial agonist properties (Wu et al., 2013). Co-treatment with the PPARγ antagonist GW9662 abolished PF11-induced adipogenesis, verifying PPARγ-dependent effects. Beyond PPARγ activation, PF11 promoted adiponectin oligomerization and secretion in mature adipocytes. Adiponectin, particularly its high-molecular-weight oligomers, functions as an insulin-sensitizing adipokine with potent anti-inflammatory and anti-diabetic properties. Enhanced adiponectin production is a key mechanism through which PPARγ agonists improve insulin sensitivity. Furthermore, PF11 inhibited obesity-linked phosphorylation of PPARγ at serine 273 by cyclin-dependent kinase 5. This phosphorylation event contributes to obesity-associated insulin resistance and adipokine dysregulation. By preventing this modification, PF11 may preserve PPARγ function and maintain insulin sensitivity in obesity and metabolic dysfunction (Figure 6).

### 3.6 Proposed mechanistic framework: a speculative multi-axis model

**FIGURE 7:**
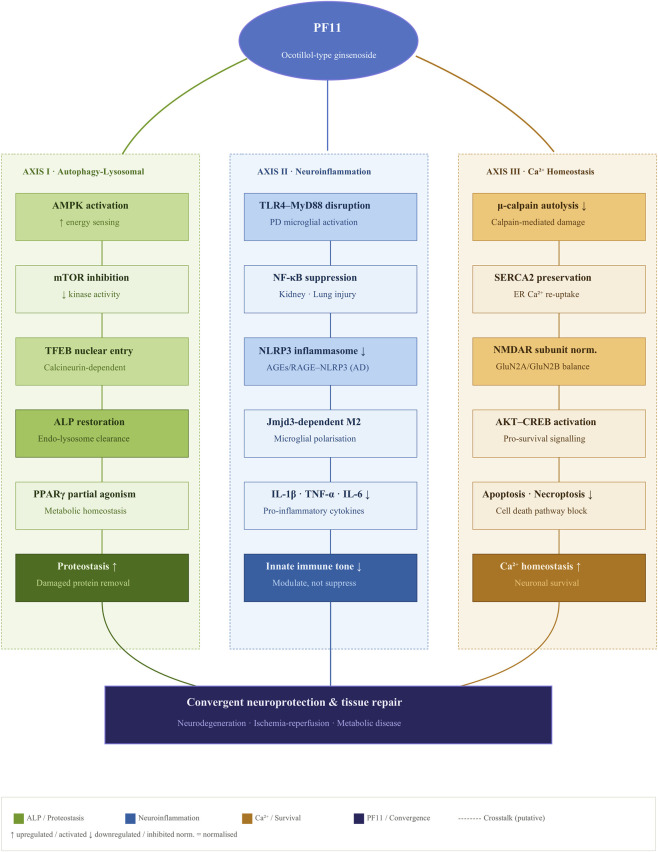
Proposed mechanistic framework of PF11 across three convergent signaling axes. Note: PF11 modulates three convergent cellular stress-response axes in preclinical models. In Axis I, PF11 activates AMPK–mTOR–TFEB signaling to restore autophagy-lysosomal pathway (ALP) function and proteostasis. In Axis II, it suppresses TLR4–NF-κB–NLRP3 signaling and promotes microglial M2 polarization, reducing pro-inflammatory cytokine output. In Axis III, it stabilizes calcium homeostasis via μ-calpain inhibition and SERCA2 preservation, and activates AKT–CREB pro-survival signaling to limit apoptotic and necroptotic cell death. Dashed arrows indicate putative inter-axis crosstalk. This framework is speculative, based entirely on preclinical data, and is intended to generate testable hypotheses. ALP, autophagy-lysosomal pathway; SERCA2, sarco/endoplasmic reticulum Ca^2+^-ATPase 2; TFEB, transcription factor EB. Sonnet 4.6.

From the evidence provided in Sections 3.1–3.5, a model of a partially overlapping triple-axis mechanism is hypothesized (Figure 7):

Axis 1 (Regulation of Cell Survival and Proliferation): PI3K/Akt/mTOR—The PI3K pathway seems to be involved in this axis, being stimulated in neurons and inhibited in cancer cells in a context-dependent manner. It is not certain whether the context dependence is based on different cellular environments of receptors rather than the effect of PF11 on PI3K itself, though its mechanism remains unknown.

Axis 2 (Resolution of Inflammation): NF-κB suppression/Nrf2 activation—This hypothesis has been proven in several cell types and pathologies, providing more convincing support for engagement in this pathway than the other two axes. Nevertheless, no binding studies or chemical proteomics analysis of the key molecular target in this axis for PF11 has been performed.

Axis 3 (Quality Control of Mitochondria): PINK1/Parkin/mitophagy—This pathway receives least convincing evidence (cardiomyocytes only), hence being the most hypothetical among all three hypotheses.

The three-component model described here is a proposed framework based on results gathered under various cell types, various organisms, various disease models, and various experimental conditions. Notably, no study has yet shown all three components being active simultaneously under one biological setting. The model cannot therefore be taken as a biological dogma, but rather as an organizing principle for directing further mechanistic studies based on hypothesis generation. In particular, targeted multiplex studies that would combine cell-type-specific and condition-specific signaling profiling through techniques such as chemoproteomics or proximity labeling are critical to making any mechanistic claims.

## 4 Pharmacokinetics

### 4.1 Absorption and bioavailability

The absorption characteristics of PF11 remain insufficiently defined in published literature, though inferences can be drawn from broader ginsenoside pharmacokinetic studies. Like other ginsenosides, PF11 is presumably associated with low oral bioavailability—a well-documented phenomenon for structurally related compounds. Pharmacokinetic investigations of ginsenosides consistently report bioavailability below 5% for both protopanaxadiol and protopanaxatriol -type ginsenosides (Leung and Wong, 2010). This poor systemic exposure arises from multiple factors: extensive first-pass metabolism, limited membrane permeability due to high molecular weight and hydrophilicity, and substantial biotransformation by intestinal microbiota (Stuurman et al., 2013; Zhang et al., 2023). PF11’s molecular structure, featuring a disaccharide moiety at the C-6 position, likely imposes similar physicochemical limitations, hindering passive diffusion across biological membranes.

The gastrointestinal metabolism of PF11 involves complex interactions with the gut microenvironment, which profoundly impacts its systemic availability. Following oral administration, ginsenosides encounter acidic conditions in the stomach, where limited hydrolysis may occur (Jeon et al., 2020; Xie et al., 2019). More significantly, PF11 undergoes extensive biotransformation by intestinal bacterial enzymes in the colon. Studies on American ginseng constituents in humans have identified multiple metabolites in biological matrices, indicating that parent ginsenosides undergo stepwise deglycosylation (Wan et al., 2016). This bacterial metabolism acts both as a barrier to parent compound absorption and a potential activation pathway, as deglycosylated metabolites often exhibit enhanced membrane permeability and biological activity compared to their glycosylated precursors. While the specific bacterial species responsible for PF11 metabolism have not been systematically identified, genera such as *Bacteroides*, *Bifidobacterium*, *Eubacterium*, and *Lactobacillus* possess β-glucosidase activity capable of cleaving ginsenoside sugar moieties (Chen et al., 2016).

Interindividual variability in gut microbiota composition introduces significant heterogeneity in ginsenoside metabolic profiles and subsequent bioavailability (Li et al., 2022). Research on related ginsenosides has shown that individuals can be classified as high-efficiency or low-efficiency metabolizers based on microbial community structure, with these differences directly correlating with compound exposure levels (Dong et al., 2017). This metabolic variability likely extends to PF11, suggesting therapeutic responses may partially depend on the functional capacity of an individual’s intestinal microbiome. Factors modulating microbiota composition—including antibiotic use, dietary patterns, and disease states—could thus markedly influence PF11 pharmacokinetics. Emerging evidence indicates that co-administration with ginseng polysaccharides may enhance ginsenoside absorption by regulating gut microbiota composition and promoting beneficial bacterial populations involved in metabolic activation (Shen et al., 2018).

Despite PF11’s demonstrated therapeutic effects in preclinical models at doses of 3–30 mg/kg, systematic pharmacokinetic parameters (Cmax, Tmax, AUC, t_1_/_2_, and clearance) remain unreported. Of note, pharmacokinetic studies of American ginseng extracts have detected various ginsenosides in plasma at nanogram per milliliter concentrations following oral administration (Choi et al., 2020; Li et al., 2019). The key pharmacokinetics study by Li et al. (2019) showed that PF11 displays absolute oral bioavailability at only around 1.2% in rats, suggesting that systemic exposure upon oral administration is negligible. Therefore, the value obtained suggests that PF11 is among poorly absorbed triterpenoid saponins and supports the notion of the severe translational problem associated with oral drug delivery.

The key paradox emerging from this finding and the available data on PF11 can be stated as follows: despite poor systemic availability via the oral route, there are several preclinical trials demonstrating pronounced therapeutic efficacy upon oral administration at the doses between 3 and 32 mg/kg. The following possible reasons may explain the paradox mentioned above. Firstly, the products of PF11 metabolism by the action of gut microflora may be more permeable and play a significant role in its pharmacological effects. Secondly, tissue-specific localization may result in sufficient local concentration.

None of these, however, has yet been directly confirmed for PF11. Notably, the existing literature does not offer any PK/PD modeling that would be able to correlate plasma levels with metabolites and therapeutic effects. In the absence of this kind of data, there is no way to prove the effectiveness of oral administration in an adequate manner. Instead, it is better viewed as a challenge to overcome during development.

### 4.2 Distribution and metabolism

Tissue distribution characteristics of PF11 represent another critical knowledge gap. While PF11 has exhibited pharmacological effects across multiple organ systems, quantitative distribution studies are lacking. Preclinical studies have demonstrated its neuroprotective effects in models of AD, PD, and cerebral ischemia—implying that PF11 or its metabolites can cross the blood-brain barrier to exert central actions (Wang et al., 2013b; Zhu et al., 2021). Similarly, its nephroprotective effects against cisplatin-induced toxicity and anti-inflammatory actions in lung tissue confirm effective distribution to these peripheral compartments (Wang et al., 2014). However, quantitative studies using radiolabeled compounds or highly sensitive analytical methods have not been conducted to establish tissue-to-plasma concentration ratios or identify organs of preferential accumulation.

The metabolic pathways governing PF11 biotransformation require comprehensive characterization. Based on the established metabolism of structurally analogous ginsenosides, PF11 likely undergoes sequential deglycosylation to generate metabolites with progressively fewer sugar residues (Chopra et al., 2023; Hu et al., 2013). The initial step involves cleavage of the terminal rhamnose moiety, followed by removal of the glucose unit, ultimately yielding the aglycone ocotillol. Phase II conjugation reactions—including glucuronidation and sulfation—may further modify both the parent compound and its deglycosylated derivatives, enhancing aqueous solubility for renal elimination. The hepatic cytochrome P450 enzyme system may also contribute to PF11 metabolism, though specific isoforms and their relative contributions remain undefined. Clarifying these metabolic routes is essential for predicting drug-drug interactions and identifying active metabolites that contribute to PF11’s overall pharmacological effects.

Excretion pathways for PF11 have not been explicitly investigated, but general ginsenoside elimination studies provide contextual insights. Ginsenosides are typically eliminated via both biliary and urinary routes, with fecal excretion predominating for compounds undergoing extensive enterohepatic circulation or remaining unabsorbed in the gastrointestinal tract. Studies on American ginseng metabolites in human biological specimens detected ginsenoside Rb1 and its bacterial metabolite compound K in plasma, urine, and feces—with fecal concentrations substantially exceeding those in other matrices (Wan et al., 2016). This pattern indicates that a significant fraction of orally administered ginsenosides escapes systemic absorption and is eliminated unchanged or as bacterial metabolites in feces. Urinary recovery of ginsenosides is generally minimal, accounting for less than 2% of the administered dose. The elimination half-life of ginsenosides varies widely based on structural characteristics, suggesting PF11 may exhibit similar temporal diversity in clearance kinetics.

### 4.3 Formulation strategies and therapeutic implications

The poor oral bioavailability of ginsenosides has spurred significant interest in developing formulation strategies to improve systemic exposure. Various approaches have been explored for structurally related compounds, including lipid-based delivery systems, microemulsions, nanoparticles, and complexation with absorption enhancers (Guo et al., 2021; Ioele et al., 2022; Ranganathan et al., 2018). Proliposomal formulations containing bile salts have increased the bioavailability of certain ginsenosides by over 180% by facilitating their conversion to mixed micelles in the intestinal environment (Hao et al., 2016). Similarly, self-microemulsifying drug delivery systems enhance ginsenoside permeability through improved solubilization and membrane penetration (Midha et al., 2017). Applying these advanced pharmaceutical technologies to PF11 could substantially enhance its therapeutic accessibility, though systematic optimization studies remain unreported.

A critical analysis of existing literature reveals several fundamental limitations that hinder our understanding of PF11 pharmacokinetics. First, most pharmacological studies have used crude American ginseng extracts rather than purified PF11, precluding the establishment of definitive pharmacokinetic-pharmacodynamic (PK-PD) relationships for this specific compound. Second, sensitive and selective analytical methods for quantifying PF11 in biological matrices have not been widely validated or applied. Mass spectrometry-based techniques possess sufficient sensitivity to detect ginsenosides at physiologically relevant concentrations, yet few studies have specifically targeted PF11. Third, the current evidence base is predominantly derived from rodent models, with no peer-reviewed human pharmacokinetic data available for PF11. Species differences in gastrointestinal physiology, hepatic metabolism, and microbial ecology may significantly affect ginsenoside disposition, requiring cautious extrapolation of animal findings to clinical settings.

These pharmacokinetic uncertainties carry substantial therapeutic implications. Despite its presumed low bioavailability, PF11 exhibits efficacy in preclinical models, indicating that sufficient concentrations reach target sites to elicit biological responses. Distinguishing among these possibilities requires integrated PK-PD modeling, which is currently lacking. Furthermore, the relationship between PF11’s pharmacokinetics and its diverse pharmacological activities demands systematic investigation. The compound exerts effects ranging from PPAR-γ agonism and adiponectin regulation to neuroprotection and anti-inflammatory actions. It remains unclear whether these multifaceted activities are mediated by the parent compound, specific metabolites, or synergistic combinations.

From a translational perspective, PF11’s pharmacokinetic profile will ultimately determine optimal dosing strategies and administration schedules for clinical applications. Its therapeutic window cannot be established without quantitative pharmacokinetic data. Similarly, potential accumulation with repeated dosing, saturation of elimination pathways at high doses, and time-dependent changes in pharmacokinetic parameters during chronic administration all require empirical investigation. Food effects on PF11 absorption are another clinically relevant consideration, as herbal supplements are often consumed with meals, which may alter dissolution, solubilization, and first-pass metabolism.

**FIGURE 8:**
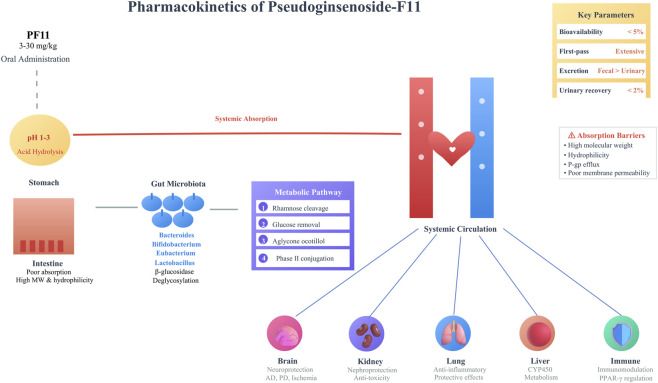
Pharmacokinetic profile and multiorgan distribution of PF11. Note: This schematic illustrates the pharmacokinetic journey of PF11 after oral dosing, including acid hydrolysis in the stomach, microbial metabolism in the intestine, key metabolic pathways (e.g., rhamnose cleavage, phase II conjugation), systemic absorption barriers (e.g., high hydrophilicity, P-gp efflux), and subsequent distribution to target organs (brain, kidney, lung, liver, and immune system) where it exerts biological effects, alongside critical pharmacokinetic parameters such as low bioavailability, extensive first-pass metabolism, and fecal/urinary excretion.

While PF11 has demonstrated promising pharmacological activities across multiple therapeutic areas, its pharmacokinetic profile remains incompletely characterized. Consistent with other ginsenosides, PF11 likely exhibits low oral bioavailability, undergoes extensive gut microbial metabolism, and achieves sufficient tissue distribution to exert biological effects in diverse organ systems. However, quantitative parameters essential for rational therapeutic development are conspicuously absent from the current literature. Addressing these knowledge gaps is critical for advancing PF11 from a promising natural product to a clinically viable therapeutic agent, enabling evidence-based dosing recommendations and supporting regulatory approval efforts for specific indications (Figure 8).

### 4.4 Quantitative assessment of translational feasibility

To make a pragmatic assessment of the translatability of PF11, a quantitative translational approach needs to be implemented. Based on the normalization to body surface area, 8 mg/kg of oral rat dose translates to ∼1.3 mg/kg HED in humans, equivalent to ∼78 mg for a 60 kg individual. However, if the oral bioavailability in humans is close to the animal level of 1.2%, then the oral dose needed to obtain comparable systemic concentration will exceed 6 g per day.

Such a high dose is impractical for use as a long-term treatment regimen unless there is a substantial improvement in oral absorption through formulation approaches. The oral dose required for achieving equivalent systemic concentrations even at five times higher bioavailability in humans is several grams per day. It is thus evident that the translatability of PF11 hinges upon formulation improvements.

## 5 Challenges and future perspectives

While preclinical studies have demonstrated promising results regarding PF11’s diverse pharmacological activities, several critical challenges need to be addressed to advance this compound into clinical practice. First and foremost, PF11’s poor oral bioavailability poses a major obstacle. Comprehensive pharmacokinetic studies in relevant animal models, and ultimately in humans, are essential to characterize absorption, distribution, metabolism, and excretion (ADME) parameters (Shikov et al., 2020; Zamir et al., 2022). These data will guide the development of appropriate dosing regimens and identify potential pharmacokinetic limitations that require pharmaceutical intervention.

Developing advanced drug delivery systems offers a promising strategy to overcome bioavailability barriers. Nanoparticle formulations, liposomal carriers, and other nanotechnology-driven delivery platforms have successfully improved the bioavailability of other poorly absorbed natural products, suggesting they may yield similar benefits for PF11 (Fernandez-Fernandez et al., 2023; Tirumala et al., 2021). Additionally, prodrug strategies, structural modifications to enhance metabolic stability, and formulation optimizations to boost dissolution rate are all worthy of exploration.

Second, the molecular targets mediating PF11’s diverse pharmacological effects need further clarification. Although multiple signaling pathways have been implicated, the direct protein targets that interact with PF11 remain incompletely identified. Unbiased approaches such as chemical proteomics, in which PF11 or its structural analogs serve as affinity probes to capture binding proteins, can uncover novel molecular targets and deepen mechanistic understanding (Kwak et al., 2024; Yao et al., 2021). Defining these primary targets will facilitate rational drug design and enable prediction of potential off-target effects.

Third, safety and toxicology studies are indispensable prerequisites for clinical advancement. While the traditional use of American ginseng provides some reassurance regarding general safety, the concentrated doses of isolated PF11 required for therapeutic efficacy demand formal preclinical safety assessments. Comprehensive toxicology studies to evaluate acute and chronic toxicity, genotoxicity, reproductive toxicity, and potential drug-drug interactions must be conducted in compliance with regulatory guidelines (McLellan and Manderville, 2017; Zhao et al., 2024).

Finally, clinical translation relies on well-designed trials to establish efficacy and safety in human subjects. Initial proof-of-concept studies in healthy volunteers can assess pharmacokinetics, tolerability, and pharmacodynamic biomarkers (Ishibashi et al., 2025; van Lier et al., 2022). Subsequent pilot trials in relevant patient populations, such as those with early-stage AD, metabolic syndrome, or individuals undergoing cisplatin chemotherapy, can provide preliminary evidence of therapeutic value. Successful completion of these studies will justify larger, definitive clinical trials.

## 6 Conclusion

PF11 is a novel ginsenoside compound that exhibits a unique structural characteristic and pharmacological actions in diverse areas such as neurology, inflammation, metabolism, and organ protection. Nevertheless, there is an imbalance in the existing literature, which predominantly focuses on *in vivo* mechanistic and therapeutic applications of PF11 while lacking sufficient data regarding PK studies, toxicological evaluation, and translational validation. One notable problem lies in the discrepancy between the oral efficacy of PF11 and its very low oral bioavailability. This calls for the urgent need to combine PK and PD in the development process as well as the design of new formulations.
